# The Influence of Genetic Variation on Social Disposition, Romantic Relationships and Social Networks: a Replication Study

**DOI:** 10.1007/s40750-018-0101-8

**Published:** 2018-08-29

**Authors:** Eiluned Pearce, Rafael Wlodarski, Anna Machin, Robin I. M. Dunbar

**Affiliations:** Social & Evolutionary Neuroscience Research Group, Department of Experimental Psychology, University of Oxford, Anna Watts Building, Radcliffe Observatory, Woodstock Rd, Quarter, Oxford, OX2 6GG UK

**Keywords:** Oxytocin, Beta-endorphin, Dopamine, Serotonin, Testosterone, Vasopressin

## Abstract

**Objectives:**

Sociality is underpinned by a variety of neurochemicals. We previously showed, in a large healthy Caucasian sample, that genes for different neurochemicals are typically associated with differing social domains (disposition, romantic relationships and networks). Here we seek to confirm the validity of these findings by asking whether they replicate in other population samples.

**Methods:**

We test for associations between the same 24 Single Nucleotide Polymorphisms (SNPs) and measures of sociality as previously, in two smaller independent samples: Caucasian individuals with histories of mental illness (subclinical sample) (*N* = 140), and non-Caucasian individuals (*N* = 66). We also combined the relevant SNPs and social measures into 18 distinct neurochemical/social domain categories to examine the distribution of significant associations across these.

**Results:**

In the subclinical Caucasian sample, we confirm previous associations between (i) specific oxytocin and dopamine receptor gene SNPs and sexual attitudes and behavior, and (ii) two SNPs associated with dopamine receptor 2 and feelings of inclusion in the local community. In the non-Caucasian sample, we replicate the previous association between an oxytocin receptor SNP and anxious attachment. More generally, chi-squared tests indicated that the distribution of significant associations for each neurochemical across the three social domains did not differ significantly between the original sample and either of the new samples, except for oxytocin in the non-Caucasian sample.

**Conclusions:**

These results corroborate both the SNP-specific and broader neurochemical associations with particular facets of sociality in two new populations, thereby confirming the validity of the previous findings.

**Electronic supplementary material:**

The online version of this article (10.1007/s40750-018-0101-8) contains supplementary material, which is available to authorized users.

## Introduction

Social network membership allows individuals to access information and resources that are essential for survival, suggesting that maintaining adequate social support has been imperative throughout human evolution (e.g. Adams, Madhavan, & Simon 2002; Cashdan 1985; Pearce & Moutsiou 2014; Whallon 2006). Indeed, it is becoming increasingly apparent that the presence of sufficient numbers of supportive social relationships has direct implications for individual health and wellbeing (Cacioppo & Cacioppo 2014; Eisenberger 2013; Holt-Lunstad et al. 2010; Umberson and Montez 2010). Enjoying positive social relationships has been linked to reduced risk of falling ill, faster recovery from ill-health and surgery, and greater longevity, as well as decreased likelihoods of exhibiting anti-social behavior, developing addictions or suffering from psychopathologies (Delvecchio et al. 2016; Eaves et al. 2010; Schindler and Sack 2015). The degree of social support an individual receives also impacts the survival of their offspring (Adams et al. 2002). Understanding the factors underlying individual differences in sociality therefore has critical consequences for promoting health and wellbeing.

A growing body of research suggests that an individual’s genetic profile plays a key role in their social cognition and behavior. For instance, the capacity to empathise have been linked to specific gene variants in the *OXTR* oxytocin receptor gene (Rodrigues et al. 2009; Lucht et al. 2013). Follow up work has found that whereas cognitive empathy, which involves being able to take another person’s perspective and to identify the emotions they are expressing, has been linked to variation in variation in the *AVPR1A* vasopressin receptor gene, while *OXTR* oxytocin receptor gene variation was linked specifically to emotional or affective empathy, or feeling how another feels (Uzefovsky et al. 2015), although this contrasts with Rodrigues et al.’s (2009) finding that *OXTR* is linked to both facets.

In addition, disparities in another aspect of social disposition, how secure an individual feels in their close relationships, have also been tied to genetic variation. The dopamine receptor 2 gene (*DRD2*) has been linked to individual variation in anxious attachment, which reflects the degree to which someone fears abandonment and rejection, and doubts their desirability as a partner (Gillath et al. 2008). In contrast, variation in avoidant attachment, which gauges how much an individual prefers to distance themselves from close relationships and prefers self-reliance to interdependence, has been linked to a polymorphism of the serotonin receptor gene *HTR2A* (Gillath et al. 2008). Moreover, variation in the *OPRM1* mu-opioid receptor gene, to which β-endorphin binds, has been found to moderate the effect of early maternal care on adult levels of fearful attachment (Troisi et al. 2012). Attachment style is linked to an individual’s response to social rejection, and variation in the latter trait has also been linked to *OPRM1* variation (Way et al. 2009). Furthermore, an *OPRM1* variant has been tied to differences in the degree of pleasure someone feels in response to social interaction (Troisi et al. 2010).

As well as affecting aspects of social disposition, genes have also been found to play a role in sexual relationships. For example, *OXTR* variants have been linked to relationship quality and pairbonding behavior in women (Walum et al. 2012), and *AVPR1A* has been linked to partner bonding, relationship status and perceived marital problems in men (Walum et al. 2008). Moreover, age at first sexual intercourse has been found to be associated with *AVPR1A* in both men and women, and the tendency to have children at a younger age in women has been tied to variation in *OXTR* (Prichard et al. 2007). In addition, an *OPRM1* SNP was found to predict speed dating success in women, whereas a *HTR2A* polymorphism was linked to dating success in men (Wu et al. 2016). Variation in the androgen receptor gene *AR* influences the degree of increase in circulating testosterone after interactions with young women, and such physiological differences might translate into behavioural ones (Roney et al. 2010).

As well as influencing social behaviour at the level of sexual dyads, there are hints that genes may also affect social engagement in wider social networks. Creswell et al. (2015) demonstrated that the rs1042778 SNP of *OXTR* influenced individual differences in negative affectivity and inhibited sociality, which in turn influenced how much social support an individual received and the size and diversity of their social network. In other words, *OXTR* variation indirectly influenced individuals’ social networks through impacting on particular dimensions of their social dispositions. Overall, it is clear that genetic variation plays a role in many aspects of sociality.

Despite the accumulating evidence of genetic influences on human sociality, until recently there were two major limitations in the literature. One has been a near-universal focus on the sociocognitive effects of genetic variation in one, or at most two, neurochemicals, ignoring the complexity of numerous interacting neural systems. The second has been a tendency to define sociality either too narrowly (by concentrating on a single social trait such as empathy) or too broadly (by lumping many diverse aspects of sociality together). To address this overly simplistic approach, Pearce, Wlodarski, Machin, & Dunbar (2017) reported on a study of 757 Caucasian adults with no history of mental illness, which simultaneously examined the associations of nine candidate receptor genes for six neurochemicals widely accepted as playing a role in sociocognitive processes: oxytocin, vasopressin, β-endorphin, testosterone, dopamine, and serotonin. The genetic associations of these genes were examined across three distinct, though interacting, domains of human sociality: ‘disposition’, ‘dyadic bonds’ (in romantic relationships) and ‘wider social networks’ (beyond the dyad) (Pearce et al. 2017).

By looking at candidate genes linked to six different neurochemical systems, we are able to examine whether these interacting systems each play a part in all aspects of sociality, or whether different systems have more ‘specialised’ roles in particular facets of sociality but not others. Previous studies, which have examined only one or two neurochemicals at a time, do not allow the relative strength of influence on different dimensions of social cognition and behaviour to be assessed, either within a neurochemical system (for instance, is oxytocin function most strongly related to sexual relationships or more broadly to social dispositional traits such as empathy?) or between neurochemical systems (for example, is dopamine or β-endorphin most strongly related to variation in wider social networks?). Moreover, over-focusing on single aspects of sociality prevents investigation of how these fit together. For example, if a particular SNP is linked to both disposition and social network size, this generates a testable hypothesis regarding possible mediation effects. Furthermore, there is evidence supporting the idea of cumulative risk, indicating that the effects of multiple SNPs need to be considered in tandem (Schneiderman et al. 2014). Tackling multiple neurochemical systems and social domains simultaneously improves on past approaches by facilitating exploration of these issues.

The findings of Pearce et al. (2017) suggested that receptor gene variation for each neurochemical had its own sphere of influence, which differed between neurochemicals. For instance, variation in the oxytocin receptor gene was predominantly linked to individual differences in relation to sexual relationships, whereas β-endorphin receptor variation was most strongly linked to social disposition, and dopamine receptor variation with wider social networks beyond the dyad (Pearce et al. 2017).

To build on these previous findings, we conducted the same analyses on two smaller independent samples from genetically and clinically distinct populations, the data for which were collected at the same time as those reported in Pearce et al. (2017). Previous studies have suggested that genetic associations with social measures might differ between clinical and healthy samples (Costa et al. 2009), so we sought to test whether the results found in the healthy sample would be replicated in a sample who reported a history of mental illness. Additionally, given the apparent existence of ‘flip-flop’ alleles that have different effects in different ethnic populations (Lin, Vance, Pericak-Vance, & Martin 2007), we also tested whether our original findings would be replicated in a sample of non-white participants. To isolate the effects of mental health profiles and ethnic background, we focused on non-white participants with no history of mental illness.

The crucial question under investigation relates to the role that different genes and neurochemical systems play in human sociality. An issue with relying solely on SNP-level analysis is an overemphasis on details that can mask overarching trends, for example relating to interactions between different neurochemical systems. Equally, combining results obtained using different measures that nonetheless relate to the same underlying constructs, such as behaviour in sexual relationships, will arguably give a more realistic model of sociality than focusing on individual measures. In other words, some degree of simplification is necessary in order to maintain explanatory power. Consequently, as well as testing for SNP-specific associations with particular social measures, we also examine broader trends in relationships between genetic variation in the different neurochemical systems (i.e. combining the SNPs related to each specific neurochemical) and the three main domains of sociality (combining related measures into the domains of disposition, sexual relationships, and wider social networks). However, by looking at six neurochemical systems and three domains simultaneously, we improve on the overly simplistic approaches used previously. Combining these two levels of complementary analysis thus provides an optimal approach to understanding how genetic variation in neurochemical systems translates into individual differences in social cognition and behaviour.

## Methods

### Participants

The current paper reports on the analysis of two samples. The first sample comprises participants of white ethnicity who reported that they had a history of mental illness (*N* = 140, 95 female, age *M* = 38.59 years, range = 18–75 years). We refer to this as the ‘subclinical’ sample, since sample members were not currently suffering from psychopathologies but had done so in the past. The second sample consists of participants who were of non-white ethnicity (4 Black African, 12 Chinese, 20 Indian subcontinent, 6 Other Asian, 16 Mixed Black Caribbean, and 8 Other) and reported no history of mental illness (*N* = 66, 33 female, age *M* = 31.27 years, range = 18–75 years). All participants were screened to exclude anyone who habitually used recreational drugs, was on drug replacement therapy such as methadone or was currently taking medication for depression, psychosis or anxiety. The 11 participants who were non-white and reported a history of mental illness were excluded. We compare these new samples to previously published data collected at the same from a sample of Caucasians with no history of mental illness (see Pearce et al. 2017).

### Procedure

Saliva samples were collected using OrageneDNA collection kits and questionnaires were answered on iPads or laptops, at UK science festivals and a museum.

In the survey administered to all participants, participants were asked: ‘Do you have a history of mental health issues such as depression, psychosis (e.g. schizophrenia) or anxiety?’ and ‘Please tell us the names of the mental health condition with which you’ve been diagnosed’. The frequencies of the broad categories reported by participants are given in Table 1 for reference. We included individuals who reported ever having had an episode of mental illness, regardless of how long ago this was. Although different psychopathological conditions are likely to have different underlying constellations of causes, here we focus on genetic associations with social phenotypes across different diagnostic categories.

**Table 1:** The frequencies of different self-reported mental illness conditions for the subclinical sample

Condition	Frequency	Percent
Addiction	1	0.7
ADHD	1	0.7
Anxiety-related	37	26.4
Borderline personality disorder	1	0.7
Condition not given	15	10.7
Depersonalisation	1	0.7
Depression (including post-natal)	76	54.3
Dissociative identity disorder	1	0.7
Eating disorder	2	1.4
OCD	1	0.7
Psychotic episode	1	0.7
PTSD	3	2.1
Total	140	100.0

Participants were asked to select their ethnicity from a standard list. ‘White British, White (other) and White Irish were groups together as ‘white’ ethnicity (Pearce et al. 2017) and all other ethnicities were coded as ‘non-white’. Only non-white participants without a history of mental illness are included here.

To measure dispositional empathy we used the revised Reading the Mind in the Eyes (RMET) test (Baron-Cohen et al. 2001), which measures how well individuals can interpret others’ emotions. This measure comprises 36 photos showing the eye region of different faces expressing different emotions. Each photo was presented separately. Participants were asked to identify the correct emotion from a choice of four words, such as ‘ashamed’, ‘nervous, ‘suspicious’ or ‘indecisive’. The total number of correct responses gives a score: higher scores indicate greater cognitive empathy. In addition, we used the self-report Empathising Quotient (EQ) scale (Wakabayashi et al. 2006), which comprises 39 items, 6 of which are reverse-scored, such as ‘I can easily tell if someone wants to enter a conversation’ and ‘I find it difficult to judge if something is rude or polite’ (reverse-scored). Participants are asked to rate the extent to which each statement accords with their view of themselves, on a 4-point scale anchored as ‘strongly disagree’, ‘slightly disagree’, ‘slightly agree’ and ‘strongly agree’. Mean scores are taken to take account of missing responses and higher scores indicate greater self-reported empathy.

Dispositional attachment styles (avoidant and anxious dimensions) in close friendships (rather than romantic/sexual relationships) were measured using the short-form Experiences of Close Relationships scale (Wei et al. 2007). This is composed of 12 items (4 reverse scored), 6 for each of the two dimensions, including items such as ‘My desire to be very close sometimes scares people away’ (anxious) and ‘I am nervous when close friends get too close to me’ (avoidant). Responses can range across a 7-point scale from ‘strongly’ disagree’ to ‘strongly agree’. Higher scores indicate higher levels of Anxious and Avoidant attachment respectively.

To examine the effect of genetic variation on dyadic relationships we measured general attitudes and behaviors in relation to sexual relationships using the revised Sociosexual Orientation Inventory (SOI-R) (Penke & Asendorpf 2008). This inventory is made up of three sections, with a total of 9 items (three in each section), and higher combined scores indicate that an individual is more promiscuous and willing to participate in short-term sexual relationships. The first section asks how many sexual partners an individual has had with respect to different scenarios: only once, without being in a committed relationship, and in the past 12 months. Participants are required to pick one of 9 categories demarcated as single values for 0 through 4, then as combined categories 5–6, 7–9, 10–19 and 20 or more. The second section comprises three statements, such as ‘sex without love is OK’, for which the participant is asked to rate the extent of their agreement on a 9-point scale anchored at ‘strongly disagree’, ‘neutral’ and ‘strongly agree’. One of these statements, ‘I do not want to have sex with a person until I am sure that we will have a long-term, serious relationship’, is reversed-scored. The final section asks participants to rate three questions regarding the frequency of fantasies and sexual arousal on a 9-point scale ranging from ‘never’ to ‘at least once a day’.

In addition, if individuals were in a relationship at the time of completing the survey, they were asked to complete the Relationship Assessment Scale (RAS) to provide an index of relationship quality, with higher scores indicating greater relationship satisfaction (Hendrick 1988). The original measure is made up of 7 questions, 2 of which are reverse scored, which participants are asked to answer on a 3-point scale with anchors that vary between items. For example, ‘how well does your partner meet your needs?’ is answered from a choice of ‘poorly’, ‘average’ or ‘extremely well’. However, to keep consistency with the form of other measures in the survey, we rephrased each question as a statement, such as ‘my partner meets all my needs’ that was answered on a 5-point scale ranging from ‘strongly’ disagree’ to ‘strongly agree’.

To measure participants’ sociality beyond the dyad, we measured their social network size by asking them to record their relationship to individuals whom they would turn to for help and support during times of difficulty and distress, and totaling the number of individuals listed (following Roberts and Dunbar 2011, Stiller and Dunbar 2007, Dunbar and Spoors 1995, Hill & Dunbar 2003). In addition, a modified version of the visual Inclusion of Other in Self (IOS) scale (Aron et al. 1992) was used to measure a participant’s feeling of integration or closeness to their local community. This measure consists of a sequence of seven diagrams, each of which comprises two circles, which become increasingly overlapped as the scale moves from 1 to 7. The labels of these two circles were modified to ‘self’ versus ‘community’ (rather than ‘other’).

We genotyped the same 33 SNPs as Pearce et al. (2017), from 9 genes coding for brain receptors for six neurochemicals: oxytocin (oxytocin receptor gene, *OXTR*), vasopressin (vasopressin receptor gene, *AVPR1A*), β-endorphin (mu-opioid receptor gene, *OPRM1*), serotonin (serotonin receptor genes, *HTR1A* and *HTR2A*), dopamine (dopamine receptor genes, *DRD1* and *DRD2*, as well as *ANNK1*, which is downstream of the latter and is closely functionally associated) and testosterone (androgen receptor gene, *AR*). We refer to these collectively using the umbrella term ‘neurochemicals’, since dopamine and serotonin are neurotransmitters, testosterone is a steroid, and oxytocin and β-endorphin are neuropeptides.

Following pruning based on linkage disequilibrium (LD) in the sample of participants with no history of mental illness (Pearce et al. 2017), we excluded 7 SNPs from the both the subclinical and non-white samples presented here: rs2268491 (*OXTR*), rs686 (*DRD1*), rs4532 (*DRD1*), rs510769 (*OPRM1*), rs1381376 (*OPRM1*), rs10877969 (*AVPR1A*) and rs6313 (*HTR2A*). In addition to these, two SNPs were also excluded due to very low minor allele frequencies (only 4% of the Caucasian healthy sample were carriers of the minor allele: Pearce et al. 2017): rs3759292 and rs1801028. This left 24 SNPs in total: 10 *OXTR* SNPs, 2 *AVPR1A* SNPs, 5 *OPRM1* SNPs, 1 *AR* SNP, 1 *DRD1* SNP, 2 *DRD2* SNPs, 1 *ANKK1* SNP, 1 *HTR1a* SNP and 1 *HTR2a* SNP.

Participants with less than 90% coverage (that is, who had missing data for more than 10% of the SNPs) were removed, and all the SNPs had at least 95% coverage (that is, missing data for less than 5% of participants in each sample, except rs6152 in the non-white sample because this SNP was not genotyped for the first set of data collected, resulting in 5 missing participants for this SNP). Table S1 gives genotype frequencies for all SNPs for which models were run. In the subclinical sample, only rs495491 (*OPRM1*) showed a significant deviation from Hardy-Weinberg (H-W) equilibrium (*p* = 0.041). In the non-white sample, rs2228485 and rs265981 genotype frequencies were significantly different from a H-W distribution (*p* = 0.037 and *p* = 0.044 respectively).

### Individual SNP Analyses

Standard genetic analyses were run separately for each SNP for each of the social measures to test whether the previously reported results were independently replicated in the subclinical and non-Caucasian samples. Following Pearce et al. (2017), genotypic models, which are equivalent to multiple linear regressions, were run for all SNPs (except the *AR* SNP: see below) using PLINK version 1.9 (Chang et al. 2015; Purcell et al. 2007). Genotype was included as the independent variable of interest, for which participants fall into one of three categories: homozygote for the minor (lower frequency) or major (higher frequency) allele (e.g. AA or GG), or heterozygote (e.g. AG).

The models test for several different possible genetic effects. Firstly, additive genetic effects (*add*) are included as a dummy variable coded as 0, 1, 2, indicating the number of minor alleles carried by each participant. A positive coefficient for this term indicates that the dependent variable increases as a function of the increasing number of minor alleles carried. In other words, this term indicates a linear dose-response effect between the number of minor alleles carried and the phenotype. Secondly, deviation from additivity (*domdev*) is tested, for which heterozygotes are coded as 1 and homozygotes as 0. This indicates whether there is heterozygote advantage. Thirdly, the combined effect of additivity and deviation from additivity (*geno_2df*) is tested, which gives an indication of dominance effects whereby carriers of one allele, whether homozygotes or heterozygotes, differ from homozygotes of the other allele.

Since the *AR* SNP is haploid, dominance tests are inappropriate, and additive models only are reported in this case. Sex and age were included in all models to examine genetic effects independent of these demographic variables. Age and sex results are reported in Tables S2 & S3 for completeness, but are not the focus of this paper.

Insufficient distributions across sex and genotype combinations (that is, where there were zero participants in at least one sex/genotype combination category for a particular SNP) meant that PLINK failed to run models for a number of SNPs (see Table 2). For the subclinical sample, this excluded the *OPRM1* SNPS rs495491 and rs3778151, as well as the *OXTR* SNPs rs2268490, rs2254298 and rs4686302 across all three social domains. For the non-white sample, this excluded the *OPRM1* SNPs rs494491, rs3778151, and rs648893, *AVPR1A* SNP rs11174811, and *OXTR* SNP rs7632287 across all three social domains. For RAS scores only, PLINK was unable to run models for one SNP in the subclinical sample, OPRM1 rs648893, and four SNPs in the non-Caucasian sample: *OXTR* rs2268490 and rs2254298, *DRD2* rs1076560, *ANKK1* rs1800497. This is due to the fact that only participants who were in relationships at the time of the survey could answer the RAS scales, which assess the quality of that relationship, reducing the sample size to *N* = 36–37 for the non-Caucasian sample, depending on the SNP. For the subclinical sample RAS sample sizes were *N* = 133–138, depending on the SNP in question.

Candidate gene studies using multiple SNPs and dependent variables suffer from the issue of multiple testing, but conventional approaches for controlling for this are well-known to be conservative and inflate type 2 errors, and therefore are not always helpful (Johnson, Nelson, Troyer, Lautenberger, & Winkler 2010; Storey & Tibshirani 2003). Instead, multiple testing was controlled for by using the PLINK mperm function. Despite the fact that none of the genotypic models survived mperm correction, the consensus is that these corrections are likely to be overly conservative: for instance, they fail to account for dependence between tests resulting from involvement in the same metabolic pathways (Rice et al. 2008; Singer 2009). Following conventional good practice in genome-wide genetics studies, we therefore present the SNP-level analyses without corrections, but suggest caution in interpreting the fine detail of the results; the overall pattern, in contrast, is more robust.

### Combined Analyses Comparing Neurochemical Systems and Social Domains

**Fig. 1:**
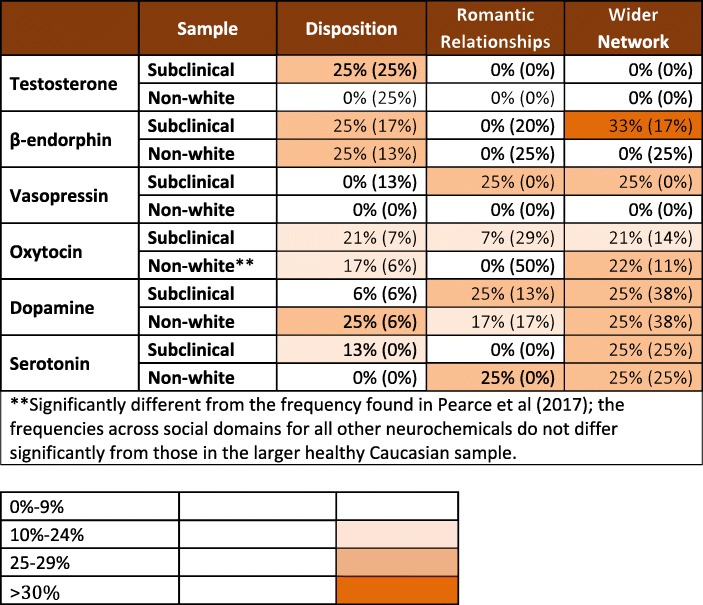
Heatmap showing the proportion of SNPs in both the subclinical and non-Caucasian samples that are significantly (*p* < 0.05) associated with the three social domains for each of the six neurotransmitter systems. Frequencies from Pearce et al. (2017), taking missing SNPs in the subclinical and non-white samples into account, are given in parentheses

As well as individual SNP-level analyses, Pearce et al. (Pearce et al. (2017) were replicated in the two new samples presented here. These follow-up analyses rely on the results of individual SNP analyses described above. However, the two sets of analyses are asking different questions. The individual SNP-level analyses test for an association between each SNP and each social measure. Here, a result is successfully replicated if a significant association between a specific SNP and a specific social measure (e.g. EQ score) found in the healthy Caucasian sample is also found in the subclinical and non-Caucasian samples. The combined analyses test whether the frequency of significant associations found in each neurochemical/social domain category (see Fig. 1) differ between the sample reported by Pearce et al. (2017) and the two samples presented here. For a conclusion of replication to be justified in this case, comparisons of observed frequencies of significant associations in each neurochemical/social domain category between the healthy Caucasian sample and either the subclinical or non-Caucasian samples would have to be not significantly different. If a significant difference between observed frequencies of associations is found between the healthy Caucasian sample and either the subclinical or non-Caucasian samples is found, this would indicate that the pattern of associations across these 18 categories differs between the original samples and those under investigation here.

Figure 1 visually represents the distributions of significant associations across the three different social domains for each neurochemical, and compares it to the original healthy Caucasian sample results. We calculated the percentage of significant associations (*p* < 0.05) in each neurochemical/social domain category given the total possible number of associations in that category, given the corresponding number of SNPs and social measures. For example, for the oxytocin system, we analysed 10 SNPs and we used four measures of social disposition, so in the oxytocin-disposition category there were a total of 40 possible associations between SNPs and social measures. The resulting matrix was coloured according to the percentage value in each cell to create a heatmap (Fig. 1): the higher the percentage of significant associations, the darker the shade of orange. This provides a quick way to ascertain the overall pattern of results for each neurochemical system in each of the three social domains. A higher percentage, represented by a darker orange, indicates that a relatively high proportion of the SNPs tested for that particular neurochemical system (combined across genes for dopamine and serotonin) were found to have significant associations with the measures in that social domain, implying a stronger relationship between individual variation in that neurochemical system and that social domain. This can be compared both across the three different social domains for each neurochemical (for example, Pearce et al. 2017 found that the oxytocin system was relatively more strongly associated with the sexual relationships domain than either social disposition or wider social networks), and between neurochemicals (for instance, Pearce et al. 2017 found that oxytocin system variation was more strongly associated with the sexual relationship domain than were the other neurochemical systems, and that dopamine and serotonin system variation was more strongly associated with wider network measures than were the other four neurochemical systems). To aid comparison between the patterns found in these two new samples and the sample reported in Pearce et al. (2017), we include the percentage values in addition to the colours.

To statistically compare the subclinical and non-Caucasian sample results to those of the original healthy Caucasian sample (Pearce et al. 2017), we ran standard 2-sample chi-squared tests comparing observed frequencies of significant genetic associations (those with *p* < 0.05) for each neurochemical across the three social domain categories between the original healthy Caucasian sample and, separately, the subclinical and non-Caucasian samples presented here. For example, to test whether distributions of significant associations for the neurochemical β-endorphin differed between the original healthy Caucasian sample and the subclinical sample, we compared the number of significant associations totaled across *OPRM1* SNPs in the ‘disposition’ category, the ‘dyad’ category and the ‘network’ category (see Fig. 1 and Table 2) between these two samples. This resulted in a two-by-three matrix (the two samples by the three social domains) for each neurochemical, to which a standard chi-squared test could be applied to compare the frequencies in the different social domains between the two samples. We conducted six chi-squared tests, one for each neurochemical. We repeated this for comparisons between the original healthy Caucasian sample and the non-Caucasian sample, yielding 12 chi-squared tests in total. A significant chi-squared test would indicate that the frequencies of significant associations differed between the two samples being compared, whereas a non-significant result would indicate that the patterns of observed significant associations did not differ statistically between the two samples. We also confirmed these chi-squared test results with Fisher’s exact tests for small sample sizes.

To ensure accurate comparisons, we excluded the SNPs that failed to produce models for either the subclinical or non-white analyses (see Table 2) from the frequencies for the healthy Caucasian sample for the respective comparisons (otherwise frequencies of significant results would have been artificially inflated in the healthy Caucasian sample compared to the subclinical or non-white samples). As a result, the comparison frequencies used for the healthy Caucasian sample given in Fig. 1 do not match Pearce et al. (2017), which in contrast could report the results for all SNPs because of the larger sample sizes involved.

**Table 2:**
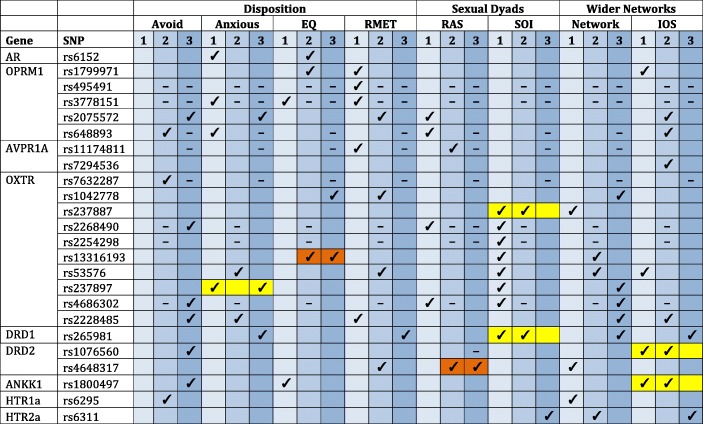
Summary of significant associations between individual SNPs and each social variable across the three samples: (1) healthy Caucasian sample, (2) subclinical sample and (3) non-white sample

**1**: healthy Caucasian sample, **2**: subclinical sample, **3**: non-white sample. Replications between the healthy Caucasian sample (1) and either of the other samples (2 or 3) are highlighted in yellow. Matching results in the other two samples are shown in orange. Minus signs indicate that PLINK failed to run models for a particular SNP for the sample indicated

## Results

### SNP-Level Analyses

We report full statistical results of all the SNP-specific associations in the electronic supplementary material Tables S2 & S3, but highlight how the results from the two new samples compare to the original findings of Pearce et al. (2017) here. We first report replicated results at the level of SNPs, before going on to discuss associations found in multiple samples for different SNPs within the same gene, as well as differences.

#### SNP-Specific Replications

In the subclinical sample, the *DRD1* and *OXTR* SNPs associated with SOI-R scores replicate the significant associations previously found in the sample of healthy Caucasian individuals (Pearce et al. 2017): *DRD1* rs265981 (*domdev x sex* effect in both samples) and *OXTR* rs237887 (*add x sex* effect in the healthy sample; *domdev x sex* effect in the subclinical sample) are significantly related to SOI-R scores in both samples, although as stated the effects are not identical (Table 2 and Table S2). Moreover, variation in the *DRD2*/*ANKK1* SNPs (rs1076560 and rs1800497) was found to be associated with IOS scores in the subclinical sample, confirming previous results (Pearce et al. 2017), although *add x sex* effects were found for healthy sample and *geno_2df* effects in subclinical sample. Furthermore, in the non-Caucasian sample, the *OXTR* SNP rs237897 was found to be associated with the anxious dimension and this replicated in the healthy Caucasian sample (Pearce et al. 2017), although an *add x sex* interaction effect was found in the non-Caucasian sample and a *geno_2df* effect in the healthy Caucasian sample.

#### Gene-Level Confirmations

In addition to replication at the level of individual SNPs, we also observed results in which the same gene, but not the exact SNP, was linked to a particular social measure in either the subclinical or non-Caucasian sample and the healthy white sample. These are not strict replications, but do indicate that variation in a particular gene might broadly influence the social measure in question.

For example, Anxious Attachment was linked to *OXTR* variation in all three samples: rs237897 (*geno_2df* effect) in the healthy Caucasian and non-white samples (as discussed above) and both the rs53576 (the more minor alleles carried the lower the anxiety) and rs2228485 (*geno_2df* effect) in the subclinical sample. In the healthy Caucasian and non-Caucasian samples, Anxious Attachment was also significantly linked to *OPRM1* variation: rs2075572 (*geno_2df*) in the non-Caucasian sample, and rs3778151 (*geno_2df* effect) and rs648893 (*add x sex* effect) in the healthy Caucasian sample. It is worth noting that both the SNPs for which significant associations were found in the healthy Caucasian sample failed to produce models in the non-Caucasian sample due to insufficient distribution across genotypic/sex categories.

In both the healthy and subclinical Caucasian samples, EQ scores were significantly associated with variation in the *OPRM1* gene: rs1799971 (positive *add x sex* effect) in the subclinical sample and rs37778151 (heterozygotes have lower scores) in the healthy Caucasian sample. Possible replication for the latter SNP could not be tested in the subclinical sample due to insufficient sample sizes across the different genotype/sex categories. RMET scores were also linked to variation in the *OPRM1* gene in both these samples: rs1799971, rs4954491 and rs3778151 showed *add x sex* interactions in the healthy sample (neither of the latter two could be tested in the subclinical sample), in contrast with rs2075572 (*domdev x sex* effect) in the subclinical sample. In addition, RMET scores were also significantly related to *OXTR* SNPs in both the Caucasian samples: rs2228284 (*geno_2df* effect) in the healthy sample and both rs1042778 (minor allele carriers have lower scores) and rs53576 (*domdev x sex* effect) in the subclinical sample.

In all three samples, *OXTR* was significantly associated with Network Size: rs237887 (*geno_2df* effect) in the healthy Caucasian sample, rs13316193 and rs53576 (both *geno_2df* effects) in the subclinical sample, and rs1042778, rs237897, rs4686302 (which could not be tested in the subclinical sample) and rs2228485 in the non-Caucasian sample. Variation in *OXTR* was also linked to IOS scores in both the Caucasian samples: rs53576 (heterozygotes showed lower scores on average) in the healthy sample and rs2228485 (*add/domdev x sex* interactions) in the subclinical sample.

#### Differences between the Original and New Sample Results

In the subclinical and non-Caucasian samples, Avoidant Attachment was linked to *OPRM1* and *OXTR* variation, as well as *HTR1a* variation in the subclinical sample and *DRD2/ANKK1* variation in the non-Caucasian sample. The latter results contrast strongly with Pearce et al.’s (2017) previous null findings regarding Avoidant Attachment.

Unlike in the healthy Caucasian sample, *AVPR1A* was not found to be associated with RMET in either the subclinical or non-Caucasian samples. *OPRM1* and *OXTR* variation was found to be associated with RAS scores in the healthy Caucasian sample, but this was not found for either of the new samples. Although the significant association between rs237887 and SOI scores was found for both the healthy and subclinical Caucasian samples, in the healthy sample an additional six *OXTR* SNPs were found to be associated with this measure but none of these showed significant effects in the two new samples (although it should be noted that small sample sizes prevented some of these SNPs being tested in the subclinical sample: Table 2). Lastly, in the healthy Caucasian sample variation in both the *DRD2* and *HTR1a* genes were found to be linked with Network Size, and *AR* variation was associated with Anxious Attachment, but these effects were not replicated in either the subclinical or non-Caucasian samples (Table 2).

There were a number of SNPs that showed the same significant association in the subclinical and non-white samples, but had not been found in the healthy white sample (Table 2). Namely, *OXTR* rs13316193 in the subclinical sample variation was significantly associated with EQ scores (positive *add x* sex effects in both samples), and *DRD2* rs4648317 was significantly associated with RAS scores in both new samples (*add x sex* effect in the non-Caucasian sample, negative additive effect in the subclinical sample: minor allele carriers had lower scores). Moreover, rs1042778 was linked to different measures of empathy in both samples (negative additive effect on RMET in the subclinical sample and an *add x sex* effect on EQ in the non-Caucasian sample), and rs2228485 was associated with attachment in both samples but to different dimensions (*geno_2df* effect on anxious attachment in the subclinical sample and *add x sex* effect on avoidant attachment in the non-Caucasian sample).

### Neurochemical/Social Domain-Level Analyses

We tested whether the pattern of significant associations for each neurochemical across social domains differed between the original healthy Caucasian sample and either the subclinical or the non-Caucasian samples. Chi-square tests revealed no significant differences in the frequencies of significant associations between subclinical and healthy Caucasian samples for any of the neurochemicals: Fig. 1. Comparisons between the healthy Caucasian and non-white samples only revealed a significant difference for the oxytocin system (Fig. 1: *χ*^*2*^ = 10.572, *df* = 2, *p* = 0.005). For all the other neurochemicals and social domains, chi-squared tests found no significant differences between the results of these two samples.

## Discussion

This study provides an important step forward in our understanding of the genetic underpinnings of human social cognition and behavior in several respects. Firstly, in contrast to most previous studies, we examine multiple neurochemical systems simultaneously, rather than in isolation, both at the level of individual SNPs and at the level of the umbrella neurochemical systems. Secondly, we incorporate multiple domains of sociality, thus providing a more realistic picture of the complexity of the human social world than studies that focus on a single social dimension. Thirdly, we provide evidence for the replication of our original findings at two levels of analysis in two independent, genetically and clinically diverse samples.

We replicated SNP-level associations found in the original healthy Caucasian sample for five SNPs (four SNPs in the subclinical sample and one in the non-Caucasian sample). Although, as is almost always the case in these kinds of analyses (Johnson et al. 2010; Storey & Tibshirani 2003), models did not survive correction for multiple testing, these replications in independent samples suggest that these associations are unlikely to be the result of inflated type 1 error. In addition, we combined SNPs into their respective neurochemical system category and demonstrated that the overall pattern of significant associations between the six neurochemicals and the three social domains did not significantly differ between the original healthy Caucasian sample and the two new samples analysed here. Overall, we can thus corroborate that the previous findings are quite robust, though more so for the overall pattern of associations across neurochemical/social domain categories than at the level of specific SNPs, even despite the relatively small sample sizes analyzed here and despite differences in mental health profile and ethnic background between samples.

In terms of broad trends, *ORPM1* variation was most strongly linked to disposition in all three samples (Fig. 1, Table 2). Given that pharmacologically blocking β-endorphin action has been shown to reduce feelings of social connection to intimates (Inagaki, Ray, Irwin, Way, & Eisenberger 2016), it would be interesting to examine further whether *OPRM1* variation, and associated individual differences in disposition, might be moderating this effect. In addition, across all three samples dopamine-related gene variation was consistently linked to engagement in the wider network: although in the non-Caucasian sample *DRD1* showed significant relationships with both personal network size and closeness to the local community scores whereas *DRD2* did so in the other two samples, this still indicates dopaminergic involvement more generally. In addition, across all three samples serotonin receptor gene variation was related to individual differences in personal networks and community connection. No significant differences were found between the frequencies of significant associations across social domains in the original sample and those in the subclinical sample for any of the neurochemicals. This was also the case for 5 out of 6 neurochemicals for the non-white sample. The exception was oxytocin*,* likely due to the surprising lack of associations with dyadic sexual relationships in the non-white sample.

In addition to replicating the broad trends of our previous research, we replicated the associations between a number of SNPs and specific social measures found previously (Pearce et al. 2017). Firstly, in both the Caucasian samples with and without a history of mental illness, sociosexual orientation was found to be significantly related to variation in dopamine *DRD1* rs265981 and oxytocin *OXTR* rs237887. The latter result showing a robust association between *OXTR* variation and sexual attitudes and behavior highlights that oxytocin seems to play a particular role in respect of dyadic relationships (Pearce et al. 2017; Prichard et al. 2007; Schneiderman et al. 2014; Walum et al. 2012). Although, to our knowledge, Pearce et al. (2017) were the first to find a direct relationship between *DRD1* variation and sexual attitudes and behaviour, it was previously found that *DRD2* variation is more strongly linked to age at first sexual intercourse when its interaction with *DRD1* is taken into account (Miller et al. 1999). Moreover, previous work has indicated involvement of *DRD1* variation in dyadic bonds in terms of maternal behaviour (Mileva-Seitz et al. 2012). In addition, *DRD1* variation seems to be associated with behaviours involving a degree of impulsivity, such as attention deficit hyperactivity disorder (ADHD) in children, and gambling in adults (Bralten et al. 2013; Da Silva Lobo et al. 2007; Misener et al. 2004). This link could explain the association with individual differences in sexual attitudes and behavior and the concomitant variation in risk-taking and orientation to immediate reward known to underlie these.

Secondly, in both Caucasian samples variation in dopamine *DRD2*/*ANKK1* (rs1076560 and rs1800497) was found to be significantly associated with feelings of integration into the community. This corroborates the novel finding of Pearce et al. (2017) that dopamine receptor variability is most strongly associated with social engagement beyond the dyad. Thirdly, the *OXTR* SNP rs237897 was significantly associated with Anxious Attachment in both Caucasians and non-Caucasians who did not report a history of mental illness (Pearce et al. 2017). Although we replicate previous null findings for attachment style and one of the most widely studied SNPs, *OXTR* rs53576 (Gillath et al. 2008; Rodrigues et al. 2009), this corroborative result for rs237897 illustrates the importance of parallel testing of a number of different SNPs for each gene, rather than focusing on a few popular ones. In support of *OXTR* variation playing a role in the Anxious Attachment phenotype, *OXTR* rs2254298 has previously been linked to anxious attachment in female adults and non-white infants (Chen, Barth, Johnson, Gotlib, & Johnson 2011; Chen & Johnson 2012). However, it is worth noting that we measured attachment styles in relation to ‘close friendships’ rather than ‘romantic relationships’ as in the standard ECR scale, and the common association with *OXTR* variation may imply some overlapping physiological underpinnings for attachment in different kinds of emotionally close relationship. However, the previous findings of Pearce et al. (2017) that the strongest association with friendship attachment is with *OPRM1* variation likely points to rather different mechanisms. This merits further investigation, particularly since the PLINK analysis was unable to run models for a number of the *OPRM1* and *OXTR* SNPs in the subclinical and non-white samples due to insufficient sample size and genotypic distributions (see Table S1), which therefore obviated tests of replication.

Despite the corroboration of the overall pattern of results as well as specific SNP associations, there were a number of interesting differences when looking at the details of the analysis. Firstly, in the subclinical sample, β-endorphin receptor gene variation had stronger effects at the network level, whereas in the healthy Caucasian sample they had their main influence at the dispositional level and in the non-Caucasian sample β-endorphin showed no effect beyond disposition (Fig. 1, Table 2). Secondly, whereas oxytocin effects were concentrated in the domain of dyadic romantic/sexual relationships in the healthy Caucasian sample (Pearce et al. 2017), in the subclinical and non-Caucasian samples the opposite pattern is observed, with a higher proportion of potential associations being significant in the disposition and network domains compared to the sexual dyad domain (Fig. 1, Table 2).

Thirdly, dopamine, while predominantly influencing network level relationships in all three samples, appears to have a greater spread of influence in the subclinical and non-Caucasian samples, with dyads and disposition showing equally strong effects, respectively. Fourthly, vasopressin also seems to behave very differently in the subclinical sample: in the healthy Caucasian population, *AVPR1A* variation yielded significant effects only in respect to disposition, but in the subclinical sample it yielded significant effects only in respect to dyadic and network level relationships. The former result for the subclinical sample chimes with previous research that has looked at repeat length polymorphisms in *AVPR1A* and found significant associations with variation in human sexual behavior (Prichard et al. 2007; Walum et al. 2008)*.* However, no effects of vasopressin receptor gene variation were observed in the non-white sample in any of the social domains, although it should be noted that only the rs7294536 SNP could be examined in this sample due to an insufficient distribution of participants across genotypic/sex categories.

Fifthly, serotonin shows an effect on sexual relationships in the non-Caucasian sample: *HTR2a* variation was linked to SOI-R scores. This corroborates suggestions that serotonin plays a role in sexual dyads: variation in *HTR1a* has previously been found to relate to whether or not the carrier is in a romantic relationship (Liu et al. 2014), which ties in well with our finding that variants in this gene are linked to the avoidant attachment style in the subclinical sample, in that dispositional attachment style is likely to influence whether or not an individual is motivated, and able, to find a partner. Despite these surface differences between the findings related to the three samples, chi-squared tests found no statistically significant differences between the frequency of positive and null results between the non-clinical Caucasian sample (Pearce et al. 2017) and either the subclinical or non-Caucasian samples presented here, excepting the result discussed for *OXTR*.

As well as these discrepancies in broad trends, a number of SNP-specific associations presented in Tables S2 and S3 differ from those reported previously by Pearce et al. (2017) but are shared between the subclinical and non-Caucasian samples. For instance, our finding that rs4648317 predicted relationship quality in both the subclinical and non-Caucasian samples supports the suggestion that *DRD2* influences behavior relating to romantic relationships: previous findings have suggested that variation in this gene is tied to ‘loving styles’ (Emanuele et al. 2007) and age at first sexual intercourse (Miller et al. 1999). Moreover, the association between *HTR2a* variation and individual differences in social interaction beyond the dyad found in both present examples (IOS in non-whites and network size in the subclinical sample) has no precedent, since in the original healthy Caucasian sample it was *HTR1a* that was linked to network size and no other measure, and *HRT2a* showed no significant associations (Pearce et al. 2017). However, the serotonin findings in the current samples add to the suggestion that genetic variation in both the dopamine (Pearce et al. 2017) and oxytocin (Creswell et al. 2015) systems may have repercussions beyond one-on-one dyadic interactions.

Although, overall, the differences between the samples are modest, it is worth exploring possible explanations for the differences that were identified. The more prosaic explanation is that they are simply spurious results arising from the small sizes of the two new samples. A more interesting alternative, however, is that, as suggested in the Introduction, these differences point to interactions between genes and other individual differences – in this case psychopathology or ethnocultural factors - manifesting in the social phenotype. Genetic influences are, of course, part of a web of interrelated factors that include the individual’s social and developmental environment. For example, an individual is more susceptible to experiencing negative consequences of an unsupportive childhood environment if they carry a particular version of specific genes (Brüne 2012; Salo et al. 2011; Troisi et al. 2012). Moreover, in some circumstances the greater the number of these ‘risky’ gene variants an individual carries, the worse their outcome (Schneiderman et al. 2014). It is clear, then, that specific genetic influences cannot be studied in isolation: other differences between individuals may also have strong effects.

For example, evidence that psychopathology may interact with gene expression to influence sociality comes from a study which found that *OXTR* variation is linked to different dimensions of attachment style in unipolar patients only, and not in healthy controls (Costa et al. 2009). These findings may indicate that, in a similar way to childhood environment in the example above, psychopathology can interact with variation in some genes to yield a symbiotic influence on social cognition and behavior. Other neurogenic differences between cases and controls may also modify the expression and function of the genes under study. For instance, there is increasing evidence of an epigenetic influence on social cognition and behavior (individuals with lower oxytocin gene methylation display more secure attachment styles: Haas et al. 2016), such that environmental or physiological feedback loops associated with mental ill-health might impact on gene expression and resulting social phenotypes. In other words, some associations between particular gene variants and specific aspects of human sociality may only manifest in individuals predisposed to psychopathology.

In addition, ethnocultural differences may also interact with genotypes to influence the social phenotype. For example, a series of studies have examined how individual variation in *OXTR* interacts with culture (European American versus Korean) to impact on various social outcomes. In Americans experiencing high psychological distress, individuals with GG/GA genotypes for the single nucleotide polymorphism (SNP) rs53576 reported seeking more emotional support than AA individuals, but genotype had no effect in Koreans, or in either ethnocultural group when distress was low (Kim et al. 2010). Since seeking emotional support is culturally normative in America but not Korea, it was argued that individuals with the genotype associated with inflated socioemotional sensitivity (GG) are more influenced by the culture within which they are embedded. This is supported by the fact that Korean Americans were found to behave more similarly to European Americans than to native Koreans: in a culture where seeking support is socially acceptable, G-allele carriers will seek support more often than AA individuals (Kim et al. 2010).

Similar results involving the same SNP have been found for emotional suppression, which is normative in America but not in Korea: GG Americans showed less emotional suppression than individuals with an AA genotype, but the opposite pattern was found for Koreans (Kim et al. 2011). The authors argue that this is because, in each case, the more socioemotionally sensitive GG individuals respond in accordance with cultural norms: low emotional suppression in America and high emotional suppression in Korea. Moreover, whereas Korean carriers of GG alleles for rs53576, who live in a culture where social affiliation is highly valued, have been found to have greater levels of psychological wellbeing if they are also more religious, GG Americans living in a more individualist culture show a negative relationship between wellbeing and religiosity (Sasaki, Kim, & Xu 2011).

It has also been suggested that worldwide allelic frequencies of putatively ‘socially sensitive’ genetic variants related to the serotonin and β-endorphin systems are significantly correlated with the relative degree of individualism/collectivism in each population (Way & Lieberman 2010). Overall, these findings indicate that one might expect that associations between genotype and social phenotype could be influenced by ethnocultural differences. For instance, the lack of an association between *OXTR* variation and sexual relationships in the non-Caucasian sample could be the result of cultural mores around sexual relationships masking any associations between sexual behavior, attitudes and *OXTR* variation. Larger samples will be needed to clarify this.

In sum, although not all the gene-sociality associations were replicated across all three samples, broad agreement exists, particularly with regard to the association between social disposition and *OPRM1* variation, and the finding that dopamine receptor variation especially affects engagement in wider social networks. The fact that we simultaneously examined all six major social neurochemicals, in the context of three separate social domains, and among genetically and clinically diverse populations, provides an important step into understanding how genetic differences underpin individual variation in social behavior and cognition. Our findings indicate that a number of SNP-specific associations, as well as broader patterns of associations across neurochemical/social domain categories, are observed across different samples despite differences in mental health profiles and ethnic backgrounds. This indicates that these are robust effects worthy of further investigation, including in relation to identifying at-risk individuals for targeted interventions. For example, the consistent association of *DRD2/ANKK1* and feelings of inclusion into the participant’s local community might indicate that certain variants of this gene predispose individuals towards loneliness, and knowledge of this susceptibility might help encourage the uptake of preventative measures. However, more work needs to be done to identify the risk alleles and clarify potential sex differences, since these effects comprised *add x sex* interactions in the original sample and *geno_2df* effects in the subclinical sample.

As well as replications, we also found a small number of potentially intriguing differences, suggesting that further enquiries into discrepancies between populations with different mental health profiles, and so-called ‘flip-flop’ genes that are associated with different effects in different ethnic populations, are required in relation to social phenotypes (Costa et al. 2009; Lin et al. 2007). It is worth noting that, given the heterogeneous nature of the non-white sample, it is possible that some of the null findings might have been the result of opposing flip-flop effects, which cancelled each other out.

Overall, these findings acquire additional relevance given the growing evidence that social network size and relationship quality have dramatic protective effects on mental and physical health, stress levels, wellbeing and happiness, as well as longevity and the ability to recover from illness, and thus ultimately evolutionary fitness, in both humans (Charuvastra and Cloitre 2008; Dominguez and Arford 2010; Holt-Lunstad et al. 2010; House 2001; Kana’iaupuni et al. 2005; Kikusui et al. 2006; Kim et al. 2016; Liu and Newschaffer 2011; Pinquart and Duberstein 2010; Reblin and Uchino 2008; Smith & Christakis 2008; Tilvis et al. 2012) and primates (Crockford et al. 2008; Silk et al. 2003, 2009, 2010; Wittig et al. 2008). For instance, both the similarities and differences in results between the three samples could inform screenings for preventative or treatment interventions, as well as suggest possible targets for drug development in relation to disorders affecting particular social domains. The similarities across samples suggest that screening based on the replicated associations, as discussed above in relation to *DRD2*, would be accurate across different populations. In contrast, associations only found in a particular sample, for example those with a history of mental illness, can feed into better understanding of how dispositional and environmental factors can interact with genotypes to influence intervention outcomes.

## Electronic supplementary material

ESM 1 (DOCX 148 kb)

Table S2 The *t* statistics for the PLINK models for each of the SNPs and social measures, indicating the *p*-level for significant genetic associations (for clarity of presentation, the significance levels for age and sex covariates are not shown), for the subclinical sample with a history of mental illness. (XLSX 41 kb)

Table S3 The *t* statistics for the PLINK models for each of the SNPs and social measures, indicating the *p*-level for significant genetic associations (for clarity of presentation, the significance levels for age and sex covariates are not shown), for the non-Caucasian sample. (XLSX 38 kb)

Table S4 (XLSX 94.2 kb)
