# Supplementary material for: The Influence of Genetic Variation on Social Disposition, Romantic Relationships and Social Networks: a Replication Study
Source: Adapt Human Behav Physiol. 2018 Aug 29;4(4):400–22. doi: 10.1007/s40750-018-0101-8 (PMC6190642; doi:10.1007/s40750-018-0101-8)
Supplement: Supplementary file 1 — (DOCX 148 kb) [file 40750_2018_101_MOESM1_ESM.docx]

**Electronic supplementary material:**

*The impact of genetic variation on social disposition, romantic relationships and social networks*

**Results**

**Sample 1: White ethnicity with history of mental illness**

***Disposition.*** Avoidant attachment scores were significantly associated with *OPRM1* rs648893 (*domdev**S *p*=0.013), *OXTR* rs7632287 (*add**sex *p* =0.002), and *HTR1a* rs6295 (*domdev**sex *p* =0.024). The *OXTR* SNP rs53576 was significantly associated with anxious attachment: additive *p* =0.039 (see Fig. 1 and Tables S1).

EQ scores were significantly predicted by variation in *OPRM1* rs1799971 (*add**sex *p* =0.01), *OXTR* rs13316193 (*add**sex *p* =0.018), and the *AR* SNP: *add**sex *p* =0.028). *RTME* scores were linked to *OPRM1* rs2075572 (*domdev**sex *p* =0.02), three *OXTR* SNPs (rs1042778 *add* *p* =0.02; rs53576 *domdev**sex *p* =0.048; rs228485 *add**sex *p* =0.02), and *DRD2* rs4648317 (*add**sex *p* =0.026, *domdev**sex *p* =0.04). The *OXTR* SNP rs7632287 approached significance (*domdev**sex *p* =0.053).

***Romantic/sexual relationships.*** Reported relationship quality (RAS) scores were significantly predicted by *AVPR1a* rs11174811 (*domdev**sex *p* =0.02) and *DRD2* rs4648317 (*add p* =0.038) variation (the associations with *OXTR* rs237887 and *OPRM1* rs2075572 approached significance: *add**sex *p* =0.054 for both) (see Table S1). Sociosexual orientation was predicted by variation in *DRD1* rs265981 (*domdev**sex *p* =0.024), and *OXTR* rs237887 (*add**sex p=0.032, *domdev**sex *p* =0.022), both of which were also associated with SOI-R in the sample of participants with no history of mental illness (Pearce, Wlodarski, Machin, & Dunbar, 2017).

***Wider network.*** Personal network size was significantly associated with variation in two *OXTR* SNPs (rs13316193 *geno_2df* *p* =0.013; rs53576 *geno_2df* *p* =0.029) and the *HTR2a* SNP rs6311 (*add**sex *p* =0.02). The degree of connection participants felt towards their local community (IOS scores) was linked to variation in two *OPRM1* SNPs (rs2075572 *domdev* *p* =0.027; rs648893 *geno_2df* *p* =0.01), the *AVPR1a* SNP rs7294536 (*add* *p* =0.05, domdev *p* =0.019), *OXTR* SNP rs2228485 (*add**sex *p* =0.008, domdev*sex *p* =0.032), and two *DRD2* SNPs (rs1076560 *geno_2df* *p* =0.004; *ANKK1* rs1800497 *geno_2df* *p* =0.009) (see Fig. 1, Table S1). Both the SNPs related to dopamine receptor 2 (*DRD2* rs1076560 and *ANKK1* rs1800497) were also significantly associated with IOS scores in the sample of participants with no history of mental illness (Pearce et al., 2017).

**Sample 2: Non-white ethnicity**

***Disposition.*** The *OPRM1* SNP rs2075572 was found to be significantly associated with both anxious (*add**sex *p*=0.04, *geno_2df* *p*=0.005) and avoidant (*geno_2df* *p*=0.049) attachment. The *OXTR* SNPs rs226890, rs4686302 and rs2228485 showed a significant association with the avoidant dimension of attachment (*domdex*sex p*=0.028, geno_2df *p*=0.011, and add*sex *p*=0.033 respectively), whereas rs237897 was significantly associated with the anxious dimension (*add**sex *p*=0.039). Whilst *DRD1* rs265981 variation was found to be significantly associated with anxious attachment (*add**sex *p*=0.007), the DRD2 rs1076560 and *ANKK1* rs1800497 SNPs were significantly related to avoidant attachment (*domdex**sex *p*=0.017 and *domdev**sex *p*=0.013 respectively). In terms of measures of empathy, both the *OXTR* SNPs rs1042778 and rs13316193 show significant add*sex effects on *EQ* scores (*p*=0.01 and *p*=0.047 respectively). In contrast, *DRD1* SNP rs265981 variation was significantly associated with *RMET* scores (*add**sex *p*=0.035, *domdev**sex *p*=0.001).

***Romantic/sexual relationships.*** The *DRD2* SNP rs4648317 showed a significant *add**sex interaction effect on *RAS* scores (*p*=0.006) and the *HRT2a* SNP rs6311 was found to have a significant *domdev**sex interaction effect on *SOI-R* scores in this sample.

***Wider network.*** The *OXTR* SNPs rs1042778 and rs2228485 showed significant *domdev* effects on personal network size (both *p*=0.037), whereas rs237897 and rs4686302 both showed significant *add**sex interaction effects (*p*=0.026 and *p*=0.049 respectively). The *DRD1* SNP rs265981 is significantly associated with both network size (*geno_2df* *p*=0.05) and *IOS* score (*domdev* *p*<0.001). The *HRT2a* SNP rs6311 was found to be significantly associated with IOS scores (*add* *p*=0.021).

**Table S1: Genotype frequencies for each SNP for all three samples, with SNPs that failed to produce models due to insufficient samples sizes across the sexes noted. The White sample refers to the data reported in Pearce et al (2017). An asterisk in the HW column denotes a significant deviation from Hardy-Weinberg equilibrium.**

| **SNP** | **Sample** | **Frequencies** | | | **HW** |
| --- | --- | --- | --- | --- | --- |
|  |  | **Minor allele homozygotes** | **Heterozygotes** | **Major allele homozygotes** |  |
| **rs7632287** | White | 44 AA | 283 GA | 429 GG |  |
|  | Clinical | 12 AA | 43 GA | 84 GG |  |
|  | Non-white (excluded) | 1 AA | 15 GA | 50 GG |  |
| **rs1042778** | White | 111 TT | 346 GT | 293 GG |  |
|  | Clinical | 19 TT | 62 GT | 59 GG |  |
|  | Non-white | 8 TT | 28 GT | 30 GG |  |
| **rs237887** | White | 139 GG | 374 GA | 242 AA |  |
|  | Clinical | 27 GG | 63 GA | 49 AA |  |
|  | Non-white | 16 GG | 30 GA | 20 AA |  |
| **rs2268490** | White | 11 TT | 174 CT | 565 CC |  |
|  | Clinical (excluded) | 2 TT | 34 CT | 103 CC |  |
|  | Non-white (excluded for RAS) | 4 TT | 24 TC | 37 CC |  |
| **rs2254298** | White | 11 AA | 162 GA | 579 GG |  |
|  | Clinical (excluded) | 2 AA | 30 GA | 107 GG |  |
|  | Non-white (excluded for RAS) | 2 AA | 20 GA | 43 GG |  |
| **rs13316193** | White | 106 CC | 345 CT | 285 TT |  |
|  | Clinical | 18 CC | 67 CT | 50 TT |  |
|  | Non-white | 6 CC | 28 TC | 31 TT |  |
| **rs53576** | White | 75 AA | 323 GA | 347 GG |  |
|  | Clinical | 14 AA | 63 GA | 62 GG |  |
|  | Non-white | 9 AA | 33 GA | 22 GG |  |
| **rs237897** | White | 120 AA | 333 GA | 304 GG |  |
|  | Clinical | 23 AA | 62 GA | 55 GG |  |
|  | Non-white | 12 AA | 33 GA | 21 GG |  |
| **rs4686302** | White | 14 TT | 154 CT | 589 CC |  |
|  | Clinical (excluded) | 0 TT | 38 CT | 101 CC |  |
|  | Non-white | 3 TT | 15 TC | 48 CC |  |
| **rs2228485** | White | 62 CC | 272 CT | 420 TT |  |
|  | Clinical | 10 CC | 48 CT | 81 TT |  |
|  | Non-white | 9 CC | 20 TC | 37 TT | * |
| **rs6295** | White | 173 GG | 385 GC | 195 CC |  |
|  | Clinical | 30 GG | 79 GC | 30 CC |  |
|  | Non-white | 9 GG | 31 CG | 26 CC |  |
| **rs265981** | White | 105 AA | 368 GA | 277 GG |  |
|  | Clinical | 19 AA | 76 GA | 43 GG |  |
|  | Non-white | 10 AA | 21 GA | 35 GG | * |
| **rs1799971** | White | 13 GG | 159 GA | 581 AA |  |
|  | Clinical | 4 GG | 30 GA | 105 AA |  |
|  | Non-white | 6 GG | 29 GA | 30 AA |  |
| **rs495491** | White | 58 CC | 287 CT | 411 TT |  |
|  | Clinical (excluded) | 4 CC | 62 CT | 74 TT | * |
|  | Non-white (excluded) | 3 CC | 18 CT | 45 TT |  |
| **rs3778151** | White | 28 CC | 201 CT | 524 TT |  |
|  | Clinical (excluded) | 3 CC | 40 CT | 97 TT |  |
|  | Non-white (excluded) | 0 CC | 17 CT | 49 TT |  |
| **rs2075572** | White | 132 GG | 383 GC | 283 CC |  |
|  | Clinical | 27 GG | 60 GC | 53 CC |  |
|  | Non-white | 5 GG | 21 GC | 38 CC |  |
| **rs648893** | White | 33 GG | 297 GA | 421 AA |  |
|  | Clinical | 5 GG | 58 GA | 74 AA |  |
|  | Non-white (excluded) | 1 GG | 11 GA | 54 AA |  |
| **rs1800497** | White | 25 TT | 214 CT | 515 CC |  |
|  | Clinical | 6 TT | 45 CT | 87 CC |  |
|  | Non-white (excluded for RAS) | 7 TT | 32 CT | 26 CC |  |
| **rs1076560** | White | 14 AA | 191 CA | 550 CC |  |
|  | Clinical | 4 AA | 41 CA | 95 CC |  |
|  | Non-white (excluded for RAS) | 7 AA | 24 CA | 34 CC |  |
| **rs4648317** | White | 21 TT | 196 CT | 535 CC |  |
|  | Clinical | 4 TT | 32 CT | 102 CC |  |
|  | Non-white | 7 TT | 24 TC | 34 CC |  |
| **rs11174811** | White | 12 AA | 199 CA | 544 CC |  |
|  | Clinical | 6 AA | 34 CA | 100 CC |  |
|  | Non-white (excluded) | 0 AA | 8 CA | 57 CC |  |
| **rs7294536** | White | 15 CC | 193 CT | 547 TT |  |
|  | Clinical | 4 CC | 38 CT | 98 TT |  |
|  | Non-white | 3 CC | 17 TC | 44 TT |  |
| **rs6311** | White | 144 TT | 358 CT | 253 CC |  |
|  | Clinical | 24 TT | 58 CT | 58 CC |  |
|  | Non-white | 15 TT | 29 TC | 22 CC |  |
| **rs6152** | White | 57 AA (female only 8) | 74 GA (female only 74) | 431 GG (female only 212) |  |
|  | Clinical | 6 AA (female only 2) | 19 GA (female only 19) | 87 GG (female only 51) |  |
|  | Non-white | 5 AA (female only 0) | 8 GA (female only 8) | 48 GG (female only 22) |  |

**References**

Pearce, E., Wlodarski, R., Machin, A., & Dunbar, R. I. M. (2017). Variation in the β-endorphin, oxytocin, and dopamine receptor genes is associated with different dimensions of human sociality. *Proceedings of the National Academy of Sciences*, *114*(20), 5300–5305. Retrieved from http://www.pnas.org/content/114/20/5300.abstract
